# Abnormal intrinsic brain functional network dynamics in patients with postherpetic neuralgia

**DOI:** 10.3389/fneur.2026.1683796

**Published:** 2026-02-10

**Authors:** Yutao You, Tianci Liu, Li Luo, Qingzhou Li, Yan Yang, Daibin Jiang, Baijintao Sun, Chuan Zhang, Bing Li, Hanfeng Yang

**Affiliations:** 1 Department of Medical Imaging, Dazhou Dachuan District People’s Hospital (Dazhou Third People’s Hospital), Dazhou, China; 2 Department of Radiology, Affiliated Hospital of North Sichuan Medical College, Nanchong, China

**Keywords:** dynamic functional connectivity, graph theory analysis, herpes zoster, postherpetic neuralgia, sliding time window

## Abstract

**Background:**

This study aimed to investigate the temporal characteristics of the functional connection state patterns and the topological structure of dynamic brain functional networks of patients with postherpetic neuralgia (PHN).

**Methods:**

We recruited 23 PHN patients, 45 herpes zoster (HZ) patients and 27 matched healthy controls (HC) to participate in resting-state functional magnetic resonance imaging. We applied the sliding time window analysis method and K-means clustering analysis to identify dynamic functional connectivity (dFC) variability patterns in three groups. The graph-theoretical approach was used to investigate the topological structure of brain functional networks.

**Results:**

All participants showed three types of dynamic functional connection states (state I: strongly connected, state II: sparsely connected, state III: intermediate pattern). Compared to HC, PHN patients exhibited a decreased proportion in state I (*p* = 0.024, FDR corrected) and increased proportion in state III (*p* = 0.006, FDR corrected). PHN patients dwelled longer in state III (*p* = 0.020, FDR corrected). Besides, the dynamic topological properties of PHN patients exhibited significant alterations in nodal network metrics. Compared with HC, PHN patients had an elevated nodal betweenness centrality in the right supramarginal gyrus (*p* = 0.003, FDR corrected) and a decreased nodal degree centrality in the left transverse temporal gyrus (*p* = 0.002, FDR corrected) in state I. Furthermore, the nodal betweenness centrality in the right supramarginal gyrus in state I was positively correlated with the disease duration (*r* = 0.679, *p* = 0.008).

**Conclusion:**

Dynamic functional connection states and the variance of topological organization may offer new insights into intrinsic functional activities in PHN brain networks.

## 1 Introduction

Postherpetic neuralgia (PHN) is a chronic neuropathic pain syndrome characterized by persistent electric shock-like pain, burning pain, or stabbing pain. It is the most common and most difficult-to-treat complication of herpes zoster (HZ) (1). Severe pain can cause anxiety, depression, and even suicide in PHN patients (2, 3). A meta-analysis showed that the incidence rate of HZ in China was 4.28 cases per 1,000 person-years, and approximately 5–30% of patients may develop PHN (4). PHN not only has a negative impact on individuals’ quality of life, but also imposes a huge economic burden on society (5). However, the central pathogenesis of PHN remains unclear, and its clinical efficacy is unsatisfactory. Therefore, exploring the pathogenesis of PHN is of great significance for improving PHN treatment strategies.

In recent decades, with the rapid development of various magnetic resonance imaging (MRI) technologies, MRI has been widely applied in exploring the potential mechanisms of chronic pain, particularly in understanding the neuropathological mechanisms underlying various types of neuropathic pain (6, 7). The transition from HZ to PHN is accompanied by changes in the central nervous system. Many neuroimaging studies have revealed a close association between pain migration from HZ to PHN and abnormal changes in brain structure and function in patients (8, 9). Many scholars have discovered differences in brain activity between PHN patients and HZ patients in areas such as the limbic system, temporal lobe, and parietal lobe. These changes in brain activity may be related to the transition from HZ to PHN (10, 11). Many studies on static functional connectivity (sFC) in PHN have shown that (9, 12, 13), PHN and HZ significantly affect functional connectivity within and between brain regions. During the chronic pain progression from HZ to PHN, static functional connectivity in the brain also undergoes dynamic changes in response to disease progression. These studies provide deeper insights into the central mechanisms of pain in PHN and HZ. However, all of the above studies believe that the brain’s internal activity and functional connectivity remain stable throughout the MRI scan. However, growing evidence suggests that functional activity and functional connectivity of the brain are not static but highly dynamic during scanning (14–16). The dynamic fluctuations of brain connectivity analysis could provide new insights into the central pathophysiological mechanisms of disease. Dynamic functional connectivity (dFC) analysis has been widely used in the study of painful diseases and is utilized to explore the time-varying profiles of functional connectivity (17). Numerous studies have reported abnormalities in the functional network dynamics of the brain in patients with trigeminal neuralgia, migraine, and chronic neck pain, and these changes are associated with pain-related clinical symptoms (16, 18, 19). Tu et al. also identified two dynamic functional connectivity states in patients with chronic low back pain that were associated with chronic pain and transient pain, and found that pain intensity in patients was related to different thalamocortical network dynamics (20). dFC provides new insights for a better understanding of the intrinsic functional activity of painful disorders. dFC better reflects the ability of brain to integrate information and deepens our understanding of pathophysiological mechanisms in the central nervous system.

Therefore, the present study applied blood oxygen level-dependent resting-state functional magnetic resonance imaging (rs-fMRI) based on the sliding time window analysis method to identify dFC variability patterns between PHN patients, HZ patients, and healthy controls (HC). The graph-theoretical approach was used to explore the topological characteristics of brain functional networks. The aim of this study was to understand the central pathophysiologic changes in PHN from a functional dynamic perspective.

## 2 Materials and methods

### 2.1 Participants

This study included 23 patients with PHN (5 were excluded because of poor image quality), 45 patients with HZ (8 were excluded because of poor image quality), and 27 HC matched for age and gender recruited at the Affiliated Hospital of North Sichuan Medical College between April and December 2024. Participants’ numerical rating scale (NRS) and clinical data such as age, gender and disease duration were collected before MRI scanning.

The inclusion criteria for patients with HZ and PHN were as follows: (1) Patients were between 30 and 80 years old. (2) All patients were right-handed. (3) According to the diagnostic criteria defined by the International Association for Pain Research (IAPR) (9, 21). The diagnostic criterion for HZ was the appearance of a lesion on the skin that did not subside within 2 weeks; The diagnostic criterion for PHN was the persistence of pain for more than 1 month after the recovery of HZ lesions; it was diagnosed by two consultant physicians in the pain department. (4) Patients with HZ and PHN both had pain scores of ≥4.

The exclusion criteria were as follows: (1) other pain disorders; (2) other psychiatric or neurologic disorders; (3) history of head trauma, alcohol or drug abuse; and (4) failed or poorly coordinated MRI scans.

### 2.2 MRI acquisition

Participants underwent MRI scanning on a Siemens Skyra 3.0 T scanner with a 32-channel head coil. The subjects were secured to the head with a foam sponge and instructed to remain still. First, conventional T2-weighted images were used to exclude visible brain structural abnormalities. High-resolution T1-weighted images and rs-fMRI images were subsequently obtained.

The parameters of conventional T2-weighted images were as follows: TR = 9,000 ms; TE = 93 ms; TI = 2,502 ms; FOV = 220 mm × 220 mm; matrix resolution = 256 × 256; slice thickness = 5 mm.

The parameters of high-resolution T1-weighted images were as follows: TR = 2,300 ms; TE = 3 ms; TI = 900 ms; slice thickness = 1 mm without gap; FOV = 240 mm × 240 mm; matrix resolution = 256 × 256; flip angle = 9°; and NEX = 1.

The parameters of rs-fMRI images were as follows: TR = 2,640 ms; TE = 30 ms; flip angle = 90°; FOV = 240 mm × 240 mm; slice thickness = 3 mm; slice gap = 0.6 mm; The total rs-fMRI duration was 10 min and 43 s, with a total of 240 time points.

### 2.3 Data preprocessing

rs-fMRI preprocessing was performed using the Data Processing Assistant for Resting-State (DPABI^1^) (22). The procedures were as follows: (1) conversion of raw rs-fMRI images to NIfTI standard format; (2) removal of the first 10 time points to allow the signal to reach equilibrium, and process the remaining 230 time points; (3) the remaining volumes correction for acquisition time delay between slices; (4) head motion correction; (5) functional images were spatially normalized to the Montreal Neurological Institute space (resampled voxel size = 3 × 3 × 3 mm^3^); (6) detrending was performed to remove the linear trends; (7) temporal bandpass filtering (0.01–0.08 Hz) was used to reduce high-frequency physiology noise and low frequency drift; (8) white matter, cerebrospinal fluid, and head motion nuisance variables were removed using a Friston 24-parameter model. Participants were excluded if their head motion parameters exceeded 2.5 mm translation and/or 2.5° rotation; (9) nuisance covariate regression; (10) smoothing (6-mm fullwidth half-maximum Gaussian kernel).

### 2.4 Construction of dynamic functional networks

The dynamic brain connectome analysis toolbox (DynamicBC) was applied to compute the dFC. The average time courses were extracted from 116 regions of interests (ROIs) of Anatomical Automatic Labeling atlas (AAL) to calculate the functional connectivity. The commonly used sliding time window approach was adopted to calculate dFC alterations, with a width of 30 TR slides in steps of 1 TR according to previous studies (23, 24). Pearson’s correlation coefficients were calculated across all brain region time series, resulting in a 116 × 116 matrix per window for each subject. Then, we performed fisher Z transformation to bring the data closer to the normal distribution. Finally, we calculated the standard deviation of the Z value to depict temporal fluctuations in the correlation coefficient and characterize the variability of the dFC.

### 2.5 Characteristics of dynamic functional networks

K-means clustering analysis was used to classify the functional connectivity matrix. Manhattan distance is used to measure the similarity between different time windows. The optimal number of clusters estimated using the elbow criterion is 3 (*K* = 3) (25). The dFC matrix of all subjects was clustered into three dFC states. These three states can be considered valid since each state contains at least 10 windows (24). We extracted data to quantify three temporal properties: the fractional windows (FW, the percentage of each state within the three states in all participants); the mean dwell time (MDT, the average time the subject stayed in the specific state before changing to another state); the total number of transitions (the frequency with which a subject transitioned between states). Permutation tests (permutations = 5,000) with age and gender as covariates were performed on the three temporal attributes of the three groups of participants, and the results were corrected for multiple comparisons using the false discovery rate method (FDR correction, *p* < 0.05).

We then extracted the brain networks of all subjects in each state. Permutation tests (permutations = 5,000) with age and gender as covariates as well as network-based statistical corrections (edge *p* < 0.001, component *p* < 0.05, literation = 1,000) were performed to compare differences in the brain networks between PHN, HZ and HC groups.

### 2.6 Graph theory analysis of dynamic functional networks

The graph theory network analysis toolbox (GRETNA) was used to perform dFC network analysis to evaluate their topological characteristics. Based on previous studies, the threshold range for sparsity was identified as 0.1–0.4 (with an interval of 0.01) (26, 27). Global and node attributes are then computed. The global properties include: global efficiency (GE), local efficiency (LE), small-world property (SWP). The nodal properties include: nodal local efficiency (Ne), nodal clustering coefficient (Ncc), nodal degree centrality (Dc), nodal betweenness centrality (Bc).

The time-varying variance of the network metric was computed by permutation tests (permutations = 5,000) with age and gender as covariates to explore the topological properties of dynamic brain networks (FDR correction, *p* < 0.05).

### 2.7 Statistical analysis

Statistical analyses were conducted using IBM SPSS 27.0.1 software. Kruskal-Wallis test was used to compare age differences among the three groups. A chi-square test was used to compare gender differences among the three groups. Mann–Whitney U test was used to compare NRS scores differences between HZ and PHN groups. Spearman’s partial correlation analyses with age and gender as covariates was conducted between dFC temporal properties, topological properties and NRS scores, disease duration in patients with PHN and HZ (*p* < 0.05).

## 3 Results

### 3.1 Demographics and clinical measurements

18 PHN patients, 37 HZ patients, and 27 HC were finally included in this study. There was no significant difference among the three groups in terms of age and gender. NRS scores and disease duration were higher in the PHN group than in the HZ group (*p* < 0.05; Table 1).

**Table 1:** Clinical and demographic characteristics.

Items	HC	HZ	PHN
No. of cases	27	37	18
Age	58.22 ± 6.49	60.38 ± 10.22	63.83 ± 12.25
Gender (male/female)	14/13	18/19	10/8
Course of disease (days/months)	NA	7.05 ± 3.07	2.1 ± 2.76
NRS score	NA	5.95 ± 1.97	7.17 ± 1.79

NRS, Numerical Rating Scale; The unit of disease duration was days for the HZ group and months for the PHN group.

### 3.2 Characteristics of dynamic functional networks

**Figure 1:**
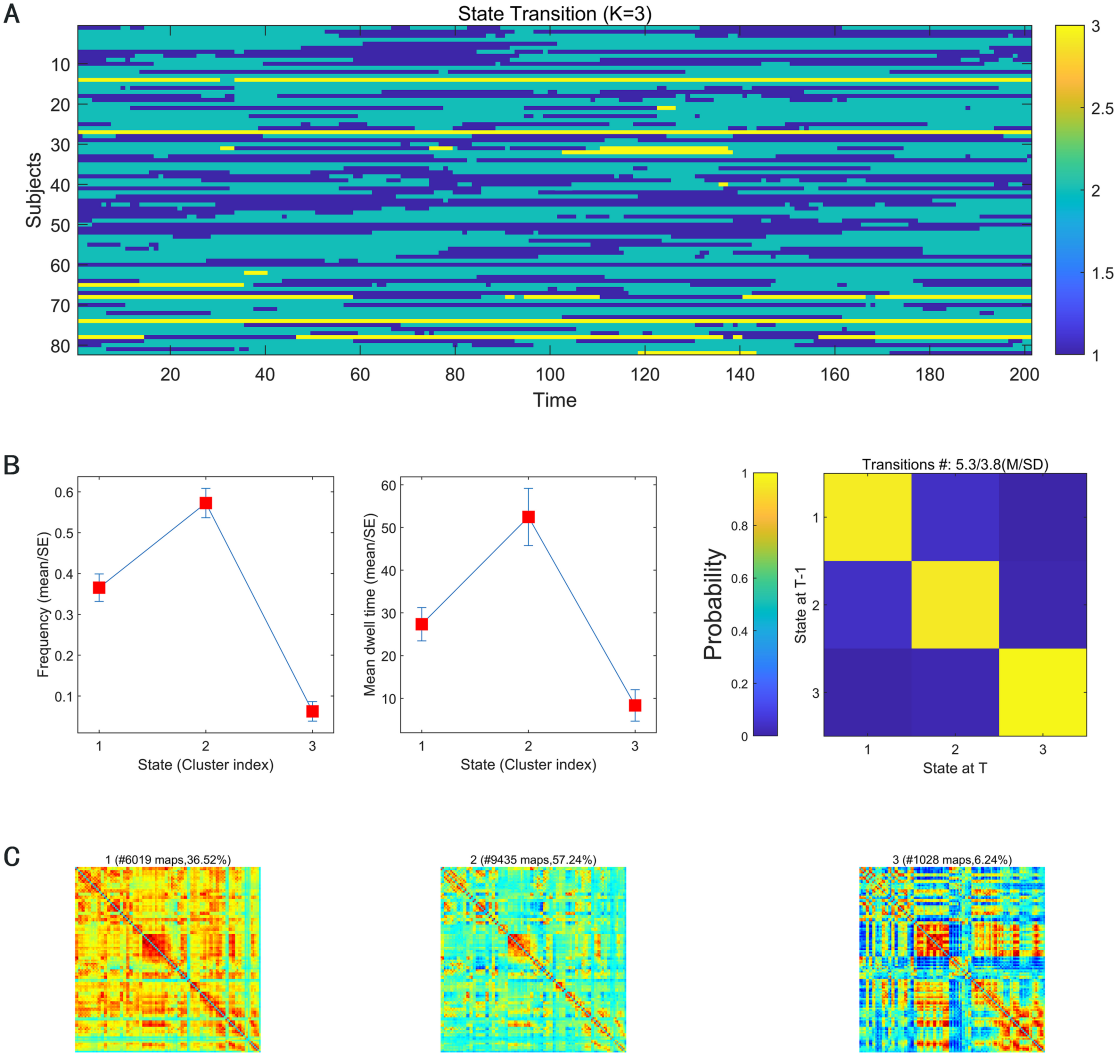
**(A)** The state-switching mode of all subjects on all sliding windows; **(B)** the mean dwell time and the probability of transition between state I, state II, and state III; **(C)** the occurrence frequencies of state I, state II, and state III.

We discerned three types of dynamic functional connection states during rs-fMRI scans among subjects (Figure 1): state I was a stronger and relatively less frequent connected state (36.52%); state II was a sparse and more frequent connected state (57.24%); state III was an intermediate pattern, but it occurs least frequently (6.24%).

**Figure 2:**
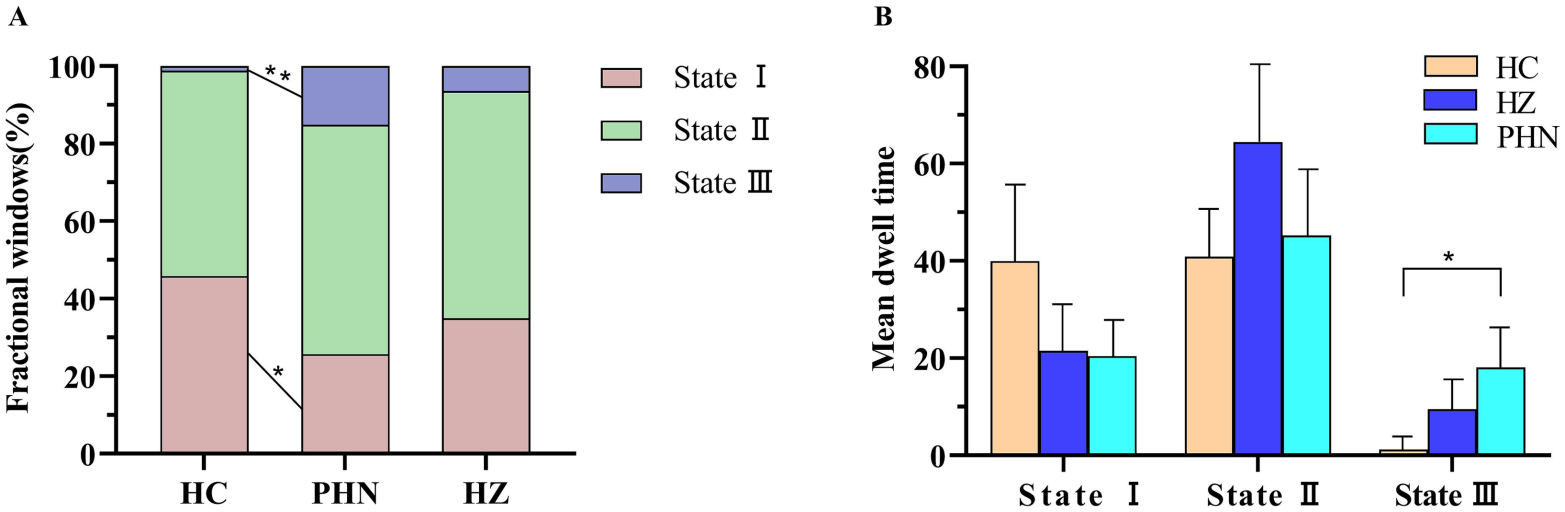
**(A)** The differences of the fractional windows between HC, PHN, and HZ groups. The fractional windows in state I (mean ± SD for PHN: 25.68 ± 21.09; for HC: 45.84 ± 31.91, *p* < 0.05) and state III (mean ± SD for PHN: 15.12 ± 31.15; for HC: 0.13 ± 0.51, *p* < 0.01) of the PHN group were significantly different from those of the HC group; **(B)** the differences of the mean dwell time between HC, PHN, and HZ groups. The mean dwell time in state III of the PHN group was significantly different from those of the HC group (mean ± SD for PHN: 18.10 ± 47.60; for HC: 0.26 ± 1.02, *p* < 0.05).

The FW in state I was significantly lower in the PHN group than in the HC group (*p* = 0.024, FDR corrected; Figure 2A). The FW in state III was significantly higher in the PHN group than in the HC group (*p* = 0.006, FDR corrected; Figure 2A). The MDT in state III was significantly longer in the PHN group than in the HC group (*p* = 0.020, FDR corrected; Figure 2B). No group differences were observed in the total number of transitions among the three states. No group differences were also observed in the brain networks between PHN, HZ and HC groups in each state.

### 3.3 Dynamic topological properties of brain networks

**Figure 3:**
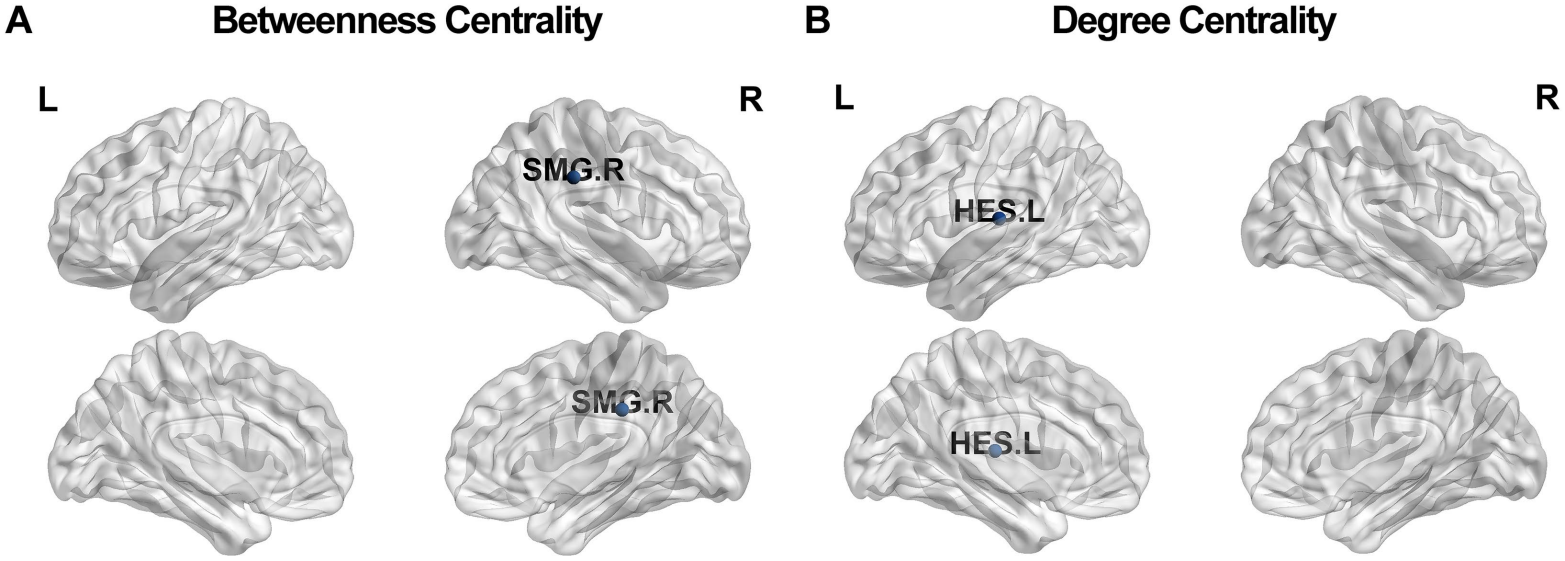
Between-group comparisons of the nodal betweenness centrality **(A)** and the degree centrality **(B)** in state I between the PHN and HC groups. The blue circles represent differences in topological properties between the PHN and HC groups. The nodal betweenness centrality in the right supramarginal gyrus (SMG. R) of the PHN group was significantly different from those of the HC group (mean ± SD for PHN: 11.17 ± 8.84; for HC: 2.81 ± 2.58, *p* < 0.01). The nodal degree centrality in the left transverse temporal gyrus (HES. L) of the PHN group was significantly different from those of the HC group (mean ± SD for PHN: 2.98 ± 2.13; for HC: 8.15 ± 3.91, *p* < 0.01).

We found that compared with HC, PHN patients had an elevated nodal betweenness centrality in the right supramarginal gyrus (*p* = 0.003, FDR corrected; Figure 3) and a decreased nodal degree centrality in the left transverse temporal gyrus in state I (*p* = 0.002, FDR corrected; Figure 3). No group differences were observed in the global efficiency.

### 3.4 Correlation analysis

**Figure 4:**
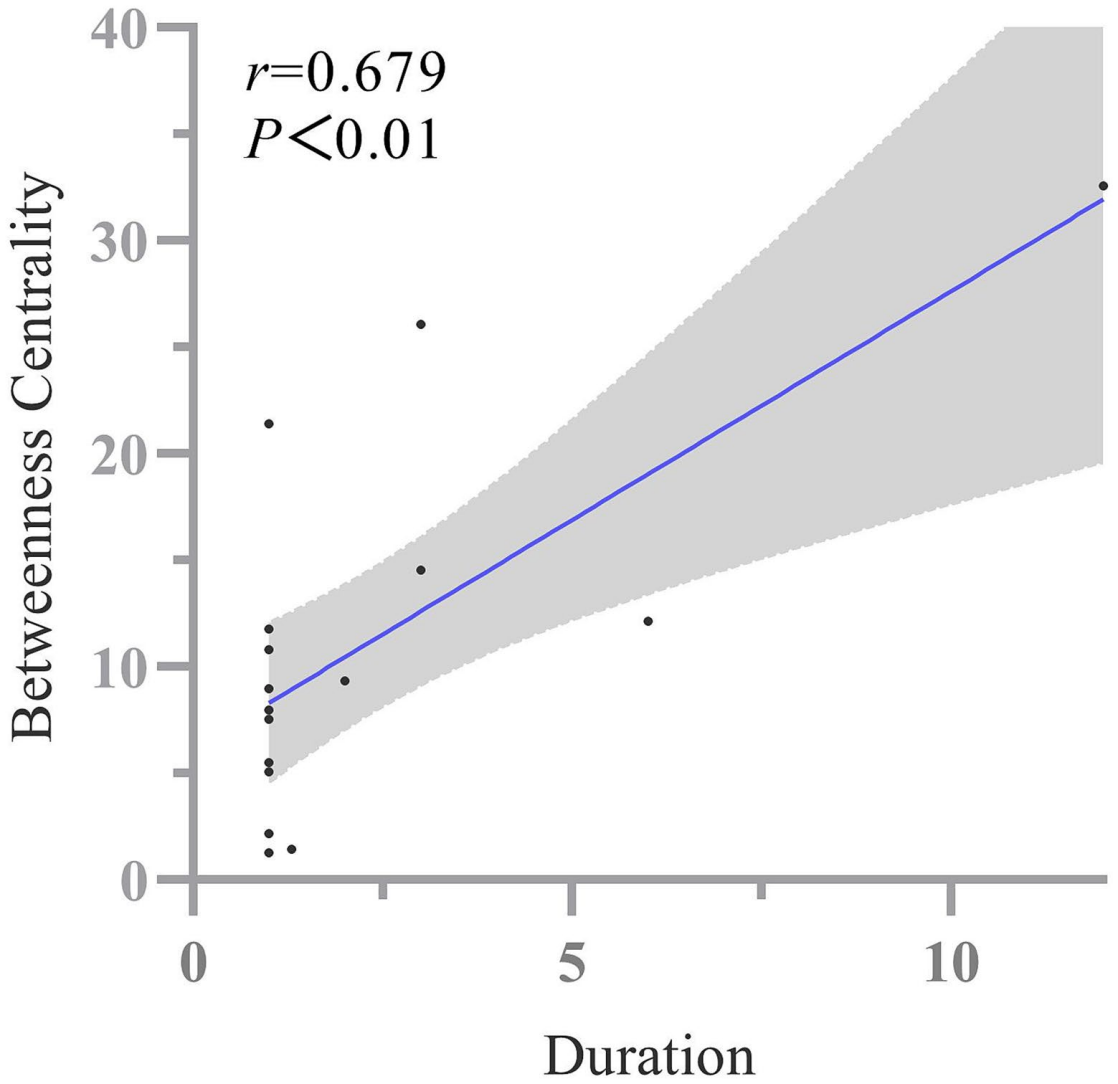
Correlation of the nodal betweenness centrality in the right supramarginal gyrus in state I with the disease duration of the PHN group.

We found that the nodal betweenness centrality in the right supramarginal gyrus in state I was positively correlated with the disease duration in PHN patients (*r* = 0.679, uncorrected *p* = 0.008; Figure 4). There was no significant correlation between the remaining indicators and disease duration, NRS scores in patients with PHN and HZ.

## 4 Discussion

Previous studies have shown that temporal characterization of dFC can elucidate central pathophysiological alterations in painful disorders from a functional dynamics perspective (17). This study explored the temporal characteristics and topological properties of dynamic functional networks of PHN and HZ patients based on the dFC approach. The results observed are as follows: (1) The PHN, HZ and HC groups all have three recurring dFC connection states (state I: strongly connected; state II: sparsely connected; state III: intermediate pattern). Compared to HC, PHN patients exhibited a decreased proportion in state I and increased proportion in state III. Besides, PHN patients dwelled longer in state III; (2) Compared with HC, PHN patients had an elevated nodal betweenness centrality in the right supramarginal gyrus and a decreased nodal degree centrality in the left transverse temporal gyrus in state I. Furthermore, the nodal betweenness centrality in the right supramarginal gyrus in state I was positively correlated with the disease duration.

Throughout the resting state, state I of strong connectivity occurred less frequently and state II of sparse connectivity occurred more frequently in all three groups of participants, suggesting that people spend more time in a sparsely connected state and less time in a strongly connected state at rest, the finding that is consistent with previous research (20, 28). Reduced functional connectivity density of multiple brain networks has been reported in patients with PHN compared to HC (9, 12). Similarly, the FW was reduced in strongly connected state I in PHN patients in our study. The reduction of the FW in the strongly connected state has been similarly found in other painful disorders and neuropsychiatric disorders, such as trigeminal neuralgia (19), cerebral small vessel disease (29) and depression (30). The strong connectivity of state I represents the presence of extensive positive connections between brain regions during this state. And the decrease in FW of strongly connected state I implies that information transfer is less efficient in PHN patients. Besides, we found that the FW and MDT of intermediate connectivity state III were increased in PHN patients compared to HC, suggesting that PHN patients are more likely to stay in the intermediate state and have difficulty switching to other states. An increase in the FW and MDT in the intermediate connection state was similarly observed in patients with knee osteoarthritis (31). Altered intermediate connectivity states in PHN patients may suggest that PHN patients have a reduced ability to flexibly switch to other states and an increased time to functional separation, leading to integration dysfunction. However, there was no difference in temporal attributes between the HZ group and the other two groups, which may be due to the shorter duration of pain in HZ, the fact that its pain was mainly driven by peripheral inflammation and alterations in the functional brain network were not yet evident.

There was no significant difference in global efficiency between PHN, HZ and HC groups, which may indicate that information transfer throughout the patient’s brain was less affected and remained stable. However, this study found significant differences in the nodal betweenness centrality and the nodal degree centrality between PHN patients and HC in state I. The nodal betweenness centrality is defined as the ratio of the number of paths passing through the node in the shortest path between all pairs of nodes to the total number of all shortest paths. The greater the nodal betweenness centrality, the greater the role of the node in the transmission of information in the network; The nodal degree centrality reflects the number of direct connections a node has to other nodes in the network, the greater the node degree centrality. The greater the number of connections to other nodes, the greater the importance of the node in the network (32, 33). In our study, PHN patients had an elevated nodal betweenness centrality in the right supramarginal gyrus in state I compared with HC, suggesting that the supramarginal gyrus may play an important role in the pathogenesis of PHN. A perfusion fMRI study has also reported elevated cerebral blood flow in the supramarginal gyrus in PHN patients (34), which is consistent with the results of the present study. Increased functional connectivity of the supramarginal gyrus has also been observed in patients with upper limb amputations (35). The supramarginal gyrus is involved in the cognitive assessment of pain and is associated with empathy and emotion recognition (36, 37). Alterations in the structure and function of the supramarginal gyrus have been found in a number of painful disorders such as migraine and spinal cervical spondylosis (38–40). Another study also found that the nodal betweenness centrality in the right supramarginal gyrus was significantly higher after repeated transcranial magnetic stimulation in PHN patients (41). These studies suggest that the supramarginal gyrus may play a key role in pain processing and perception in PHN patients. In addition, exploratory correlation analyses between these network metrics and clinical characteristics were performed, and we found that the nodal betweenness centrality in the right supramarginal gyrus was positively correlated with disease duration. Similarly, Cao et al. found reduced gray matter volume in the right supramarginal gyrus in PHN patients and a negative correlation with pain duration (8). This suggests that the supramarginal gyrus may be altered early in PHN and involved in the chronicization of pain in PHN. The transverse temporal gyrus is an important component of the auditory network (AN) and plays an important role in auditory information processing (42, 43). In this study, we found that the nodal degree centrality of the left transverse temporal gyrus was reduced in PHN patients, suggesting that auditory function may be impaired in PHN patients. Abnormalities in the node network in certain brain regions of the AN have been found in patients with spinal cervical spondylosis and lower back pain (27, 44), suggesting that the AN is unstable in patients with chronic pain. Altered functional connectivity in the AN has also been found in patients with trigeminal neuralgia and primary dysmenorrhea (19). The experience of pain is accompanied by multiple sensory inputs, such as vision, hearing, and smell. And altered AN in PHN and other painful disorders may indicate dysregulation between and within patients’ sensory-related networks (45).

## 5 Limitations

This study has several limitations. First, there is currently no consensus on the definition of PHN. The present study employed a more aggressive and traditional definition of PHN (pain persisting > 1 month after rash healing), rather than the more widely accepted definition (pain persisting < 1 month as acute herpetic neuralgia, pain persisting < 3 months as subacute herpetic neuralgia, and pain persisting > 3 months as herpetic neuralgia). Differences in PHN definitions may influence our findings. Future research should expand the sample size and further stratify the PHN group into subacute herpetic neuralgia and postherpetic neuralgia subgroups to examine differences between them. Moreover, although the sliding window length and step size in the dynamic functional connectivity analysis of this study were determined based on previous research, and the optimal number of clusters K was determined using the elbow criterion, the reliability and reproducibility of these findings require further validation. Future studies should conduct validation analyses using various analytical parameters, including different sliding window lengths, step sizes, and K-means clusters. Finally, this study employed a sliding window method based on AAL. These results should be validated by other methods, such as independent component analysis.

## 6 Conclusion

This study focuses on the variation of temporal characteristics of PHN functional connectivity from the dFC perspective. In this study, we used the K-means clustering analysis to categorize the brain network patterns of PHN patients into three states. And we found that the fractional windows were lower in the strongly connected state and the fractional windows as well as the mean dwell time were higher in the intermediate connected state of PHN. In addition, the graph-theoretical analyses were performed in this study to explore the topological properties of brain functional networks in PHN. We found that the node properties of some brain regions were altered in PHN patients and were correlated with the disease course. The results of this study contribute to a deeper understanding of the central mechanisms of pain in PHN and further emphasize the critical role of functional networks in the pathophysiology of PHN.

## Data Availability

The raw data supporting the conclusions of this article will be made available by the authors, without undue reservation.

## Footnotes

1
http://www.restfmri.net
